# Screening for Microalbuminuria in HIV-Positive Children in Enugu

**DOI:** 10.1155/2012/805834

**Published:** 2012-07-08

**Authors:** Ezeonwu Bertilla Uzoma, Okafor Henrietta Uchenna, Ikefuna Anthony Nnaemeka, Oguonu Tagbo

**Affiliations:** Department of Paediatrics, University of Nigeria Teaching Hospital Ituku Ozalla, Enugu 400001, Nigeria

## Abstract

*Background*. Human immunodeficiency virus associated nephropathy (HIVAN) is a rapidly progressive chronic renal parenchymal disease that occurs in HIV-infected individuals and manifests commonly as proteinuria, which is preceded by microalbuminuria (MA). This clinical entity is defined as a spot urine albumin of 20–200 mg/L. *Objectives*. To determine the prevalence of microalbuminuria in HIV positive children in UNTH, Enugu and compare it with that of HIV-negative children. *Methods*. A total of 154 HIV positive children aged 18 months to 14 years and 154 HIV-negative children of corresponding attributes were screened for microalbuminuria, using Micral test II strip which has a sensitivity of 90–99%. *Results*. No child among the groups (HIV positive and negative) had microalbuminuria. Majority (96.0%) of HIV-positive children had nonadvanced HIV disease at the time of the study (*P* = 0.00). About 77.3% were using HAART (*P* < 0.0001), the mean CD4 cell count of the subjects was 709.2 ± 443.9 cells/mm^3^; while 78.0% had nonsevere immunosuppression (*P* = 0.00). Furthermore, HIV-positive children with severe immunosuppression were younger and had shorter duration of treatment. *Conclusion*. Microalbuminuria may not be very common in Nigerian children irrespective of their HIV status.

## 1. Introduction

Nigeria contributes 9% to global HIV burden, and has a seroprevalence of 4.6% with 220,000 children already living with HIV infection as of 2008 [1, 2]. Human immunodeficiency virus (HIV) affects many organs including the kidney [3]. HIV renal parenchymal disease is varied and may result from direct effect of the virus on renal epithelial cells, immune-complex mediated vasculitis, hyperviscosity of blood secondary to hyperglobulinaemia, various opportunistic infections, and also drugs [4]. The commonest chronic renal parenchymal disease in HIV-positive patients is HIV-associated nephropathy (HIVAN) [5, 6], which occurs in childhood [6–9] and has been documented in Nigerian children too [9, 10]. HIVAN progresses to ESRD but if detected early, this progression can be slowed or even halted with the use of HAART [6]. The commonest manifestation of HIVAN is proteinuria, [5, 11] which has been described in HIV-positive children [6, 9] and Esezobor and colleague [9] in Nigeria documented a prevalence of 20.5% in HIV-positive Nigerian children. Microalbuminuria is a predictor of subclinical renal involvement in systemic diseases including HIVAN [3]. It precedes proteinuria [3] and has been shown to be an early manifestation of HIVAN [3]. Therefore, the detection of microalbuminuria as well as the prompt institution of HAART will reduce the morbidity and mortality associated with chronic kidney disease in children. Eke and others [10] documented a high prevalence rate of 12.0% for microalbuminuria in children. Thus screening for MA may detect early onset of renal impairment thus facilitating early treatment.

Microalbuminuria is a screening test for subclinical renal involvement in systemic diseases [3] and therefore, macroalbuminuria (proteinuria), should be excluded as it is an overt clinical manifestation of renal involvement. Other variables to be excluded include glycosuria, urinary tract infection (UTI), and alkaline pH. Also, normal urine-specific gravity should be ensured. Urine concentration is affected by both the weight and number of particles in the urine and so glucose with a high molecular weight will increase the specific gravity and thus result in concentrated urine thereby giving a false positive result [12]. Also severe glycosuria causes high-urine flow rate resulting in dilute urine and underestimation of urine albumin [13]. Proteinuria is a common observation in symptomatic UTI and may be a consequence of the reaction of the protein test pad with leukocytes and bacterial proteins in urine [14]. Studies [15, 16] have shown that urine dipstick test is negative for leucocyte esterase (LE) and nitrite may reasonably rule out UTI, so also combinations of positive LE and nitrite in urine could be used to rule in UTI. It has been shown that urine pH greater than 7.0 gives false-positive result, as the tetrabromophenol indicator in urinary dipstick is pH dependent [11, 12]. There is a direct relationship between urine specific gravity and positivity of the Micral test strip for microalbuminuria, such that high urine specific gravity increased positivity of Micral test for microalbuminuria [17]. Therefore, correction for specific gravity is needed for an accurate detection of microalbuminuria [17].

At urine albumin concentration greater than 20 mg/L, the sensitivity of Micral test II strip to microalbuminuria is greater than 95% (range of 90–99%) and specificity of greater than 80% (range of 70–90%) [11].

This study was carried out to determine the prevalence of microalbuminuria in HIV-positive and HIV-negative children using Micral test II strip, and to compare their prevalence rates.

## 2. Patients and Methods

The study was conducted at the University of Nigeria Teaching Hospital (UNTH), Enugu, one of the first generation tertiary hospital facilities, in Nigeria, catering for patients predominantly from the South-Eastern region of the country, comprising Abia, Anambra, Ebonyi, Enugu, and Imo states. Participants were enrolled from the Paediatric Infectious Disease Clinic (PIDC) and Children Outpatient Clinic (CHOP) of the hospital. Ethical approval was obtained from the Institutional Health Research and Ethics Committee. At the time of this study, there were 236 registered HIV-positive children attending the PIDC.

It was a cross-sectional descriptive study with the sample size of 308 determined using the sampling method for estimation of proportions of study population less than 10,000 [18]. A written consent and basic demographic characteristics were obtained from the caregiver/children before recruitment. The study participants were excluded if they had axillary temperature of 38.0°C [4], if urine samples were positive for proteinuria, (macroalbuminuria), glycosuria, [13] nitrite, leucocyte esterase (indicating urinary tract infection) [15, 16], and alkaline pH [11], Also, only urine with normal specific gravity (1.005 to 1.015 [12]) was used for this study [17]. From the Children Outpatient (CHOP) clinic children matched for age and sex who screened negative for HIV with the use of rapid diagnostic test kit, (DETERMINE, by ABBOT, Japan Co., Ltd., LOT 72627U100) were enrolled in the study.

To further improve the analysis of the outcome, the study participants were grouped based on the World Health Organization (WHO) age stratification for age-related CD4 values, into three groups; 18 months–35 months, 36 months–59 months, and 5 years–14 years, in the ratio of 1 : 2 : 4. This ratio represents the relative proportion of HIV-positive children attending clinic in UNTH within these age groups, similarly, the male : female ratio of 1 : 1 which was based on their gender distribution. Using this ratio, the proportion of HIV positive children enrolled in the three age groups were 22 (11 males, 11 females), 44 (22 males, 22 females), and 88 (44 males, 44 females), respectively.

On enrolment of the HIV-positive children further clinical information such as the time of diagnosis of HIV and commencement of HAART, clinical features and physical examination, Chest radiograph report, Mantoux result, sputum acid fast bacilli, and ear swab microscopy and culture results, were obtained. Blood samples from the HIV-positive children were sent to the laboratory for estimation of CD4 percent (preferred immunologic marker for monitoring HIV disease progression in children less than five years [19]), CD4 cell count (for those five years and above), and the full blood count (FBC). Partec CyFlow counter, Germany 2008, serial number 080513422 was used for the estimation of the CD4 cell count and CD4%, while Sysmex Haematolog analyzer, Europe, serial number A8206 was used for FBC. All the participants, had their urine tested further using the same sample, with Micral test II strip (Accu-Chek product of Roche Diagnostics, Australia Pty Ltd., LOT 23024338) [15, 20]. Microalbuminuria was defined as on the spot urine albumin of 20–200 mg/L [11]. The clinical assessment findings and the laboratory results were used for clinical [8] and immunological [21] staging of the subjects.

Advanced HIV disease was defined as WHO clinical stage 3 and 4, while stage 1 and 2 defined nonadvanced HIV disease [19]. Severe immunosuppression was defined as CD4 cell count of less than 200 cells/mm^3^ in those children who were five years of age or older, CD4% of less than 15% for those between 36 and 59 months, and CD4% of less than 20% for those between 18 and 35 months [21]. The full blood count result was used for WHO clinical staging; anaemia was defined as haemoglobin concentration of less than 8 g/dL, neutropenia was absolute neutrophil count of less than 1000/mm^3^, and thrombocytopaenia was platelet count of less than 50000/mm^3^[7].

The clinical information (historical, physical examination, and laboratory were recorded in the study proforma designed separately for HIV-positive and HIV-negative children and subsequently analyzed using the Software Package for Social Science (SPSS) version 15.0 for Windows. Continuous variables such as age, duration of HIV diagnosis, and duration of HAART treatment were analyzed and expressed as mean and standard deviations. Comparison of means was done using *t* test. For categorical variables, chi-squared test was used. Significant levels were set with *P* value of <0.05.

## 3. Results

A total of three hundred and twenty-five children (HIV positive and negative) who were aged 18 months to 14 years were screened. Seventeen children (5 HIV positive and 12 HIV negative) were excluded from the study after testing their urine with Multistix combi 11. Of the 17 excluded children, 16 had proteinuria. Among these 16 children; 11 were HIV-negative children and 5 were HIV positive giving a prevalence of 3.1% for the latter group. Besides the presence of macroalbuminuria/proteinuria, 13 of the 16 excluded children had other urinary findings such as urine specific gravity greater than 1.015 (8 HIV negative and 4 HIV positive), leucocyturia, and urine pH of 8 (one HIV-negative child).

Regarding the characteristics of these excluded HIV-positive children with macroalbuminuria/proteinuria, they were comparatively older than those without macroalbuminuria: 102.6 ± 56.1 versus 78.6 ± 39.1 months. Also, compared with the children who do not have proteinuria, those with proteinuria had longer duration of HIV diagnosis: 25.8 ± 13.2 versus 21.0 ± 15.9 months, shorter duration of HAART use: 24.2 ± 15.2 versus 16.1 ± 16.0 months, and low CD4 cell count: 219.4 ± 233.4 versus 711.4 ± 443.6 cells/mm^3^, *P* = 0.01. Also 3 (60%) out of the 5 children with proteinuria were immunosuppressed while 4 (80%) were on HAART.

**Figure 1:**
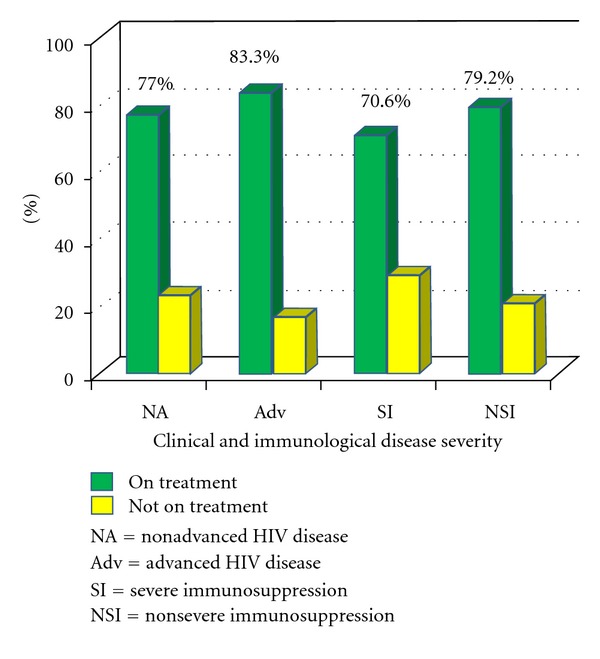
Treatment with HAART by clinical disease severity and degree of immunosuppression.

Subsequently, three hundred and eight children, comprising 154 HIV-positive (77 males and 77 females) and 154 HIV-negative children (77 males and 77 females), aged between 18 months and 14 years were enrolled. The mean age was 78.6 ± 39.1 months and 81.2 ± 45.2 months for the subjects and controls, respectively. The mean duration of HIV diagnosis for the subjects was 21.0 ± 15.9 months. Significant proportion of the subjects had nonadvanced HIV disease and nonsevere immunosuppression, and majority was on HAART (Table 1). The mean duration of treatment with HAART was 16.1 ± 16.0 months. A greater percentage of subjects in either category of clinical disease severity and immunosuppression were already on HAART treatment (Figure 1).

The range of CD4 cell count for the subjects was 47–2090 cells/mm^3^ with a mean of 709.2 ± 443.9 cells/mm^3^ and as shown in Table 2, there was a decline in the mean CD4 cell count within the age groups. Those subjects below five years of age predominated among the severely immunosuppressed, Table 3.

Subjects with advanced HIV disease were comparatively older; 103.7 ± 47.1 months versus 77.6 ± 38.6 months and had treatment for a shorter duration of time; 13.0 ± 17.8 months versus 16.3 ± 16.0 months. Similar analysis regarding the degree of severity of immunosuppression was carried out and interestingly study subjects with severe immunosuppression were relatively much younger; 69.0 ± 43.1 months versus 81.4 ± 37.7 months and have been on HAART treatment for a shorter period of time than those who were nonseverely immunosuppressed; 10.9 ± 10.2 months versus 17.6 ± 17.0 months.

The prevalence of microalbuminuria was zero percent (0%) for both subjects and controls.

## 4. Discussion

In this study, the zero percent prevalence is consistent with the finding by Leroy and colleagues [5] in France, who noted a zero prevalence of microalbuminuria in a population of 27 perinatally infected HIV-positive children. The similarities in the subject characteristics in Leroy's study with those in the present study may explain the comparability of the two findings even when a more sensitive method, nephlometry, was used for their determination of microalbuminuria [5]. A significant proportion of their subjects, whose mean age was 44 months, were on antiretroviral therapy; majority had nonadvanced disease and were not severely immunocompromised [5].

In contrast, Eke and others [10] in South-Southern Nigeria, found a prevalence of 12%. The disparity between Eke's study and the index study could be attributed to the subjects' characteristics and methodology. For those with microalbuminuria in Eke's study, mean duration of HIV diagnosis was 5.5 ± 4.6 years (all had vertical transmission), 83.3% had advanced HIV disease, none was on HAART, all were severely immunosuppressed, and (macroalbuminuria) proteinuria was not excluded [10]. The pair of these studies further illustrates the need for a stricter control of confounders in the methodology in order to improve on the specificity of the results. Their subjects' characteristics are typically the risk factors for the development of HIVAN. It may be surmised that the outcome in the determination of microalbuminuria may be related to the criteria for inclusion in subject selection. Longer duration of HIV diagnosis [22], advanced HIV disease [3, 6, 9], absence of treatment with HAART [5, 22, 23], and severe immunosuppression [3, 24] are risk factors for development of HIVAN, whose earliest manifestation is microalbuminuria [3].

Other studies [3, 8, 22, 24, 25] with varying prevalence rates for microalbuminuria were carried out in adult subjects. For example, Gupta and coworkers [25] in 2005 reported a prevalence of 9% in the 68 patients they studied, a finding that was similar to those of Szczech et al. (11%) [24], Baekken and colleagues (8.7%) [22], and Zambrano et al. (11.1%) [8]. However, a much higher prevalence of 36% was reported by Han and colleagues [3]. Besides the subjects being older, confounding factors such as hypertension, diabetes mellitus, urine with alkaline pH, and high specific gravity and UTI were not excluded in these studies.

Hypertension [3, 8, 22, 24, 25] and diabetes mellitus are associated with renal endothelial dysfunction and microvascular disease, presenting with proteinuria [24]. Proteinuria can occur in urinary tract infection, which may be a consequence of the reaction of the protein test pad with leucocytes and bacterial proteins in urine [20]. Alkaline urine gives false-positive result with urinary dipstick, as the tetrabromophenol indicator impregnated in the urinary dipstick is pH dependent [11]. The Micral test strip is sensitive to the concentration of albumin in urine [17]. There is a direct relationship between urine specific gravity and positivity of the Micral test strip for microalbuminuria, such that high urine specific gravity increased positivity of Micral test for MA [17]. The rate of false-negative results decreases with increase in urine specific gravity and false-positive result increases with increase in urine specific gravity, so that normal specific gravity is needed for an accurate detection of microalbuminuria [17]. These factors could explain the prevalence rate documented in those studies [3, 8, 22, 24, 25]. Equally pertinent in the consideration of these results is the role of these confounders in the presence of microalbuminuria irrespective of age. This leaves a poser as to the relative sensitivity of Micral test as a routine investigational/screening test for microalbuminuria in individuals whose risk factors are not known.

Use of HAART may have contributed to the zero prevalence in index study, as 77.3% were already using HAART. Treatment with HAART reverses renal epithelial changes with marked reduction in albuminuria [23]. All the subjects in Leroy's study [5] were on HAART and none had microalbuminuria whereas Eke's [10] and Han's [3] subjects were HAART naïve, and this may have accounted for their recorded prevalence of 12.0% and 36.0%, respectively.

Significant proportion of subjects in the index study had nonadvanced HIV disease and nonsevere immunosuppression, which are proven risk factors for development of HIVAN [3]. All the subjects with microalbuminuria in Eke's study [10] had advanced disease and were all severely immunosuppressed.

The prevalence of macroalbuminuria/proteinuria noted among the HIV-positive children is low in comparison to finding by other studies. Esezobor and coworkers [9], Chaparro and colleagues [6] found 20.5% and 33% prevalence rates, respectively. Our low prevalence may be attributed to some factors such as the subject selection method, as well as the characteristic of the cohorts who were observed to have essentially low risk factors. They were younger [3], had nonadvanced disease [3, 9], on HAART treatment [5, 6, 9], and had high CD4 cell count [3, 5, 6]. Furthermore relative to the HIV-negative children the difference although insignificant could also be due to the many factors which were related to the use of medication among the HIV positive group. This is known to impact on the excretion of proteinuria. However the use of the assessment method could also have played a role, it may have been possible to differentiate the protein types if a more robust method was used in the screening. Also the aetiology of the subjects with proteinuria would have been determined by renal biopsy which was not done in the subjects as it was not feasible.

It is pertinent to note that absence of HAART-naïve subjects, limited the ability of the study to assess the impact of HIV infection on the kidney. The use of albumin-creatinine ratio (ACR) or the use of nephlometry, in the determination of microalbuminuria would also improve the quality of the outcome of this study, but none was done.

The essence of the study was to determine the need for the use of the Micral test in screening of patients who may have microalbuminuria as an initial indicator of HIVAN. The results have shown that if other risk factors are eliminated, microalbuminuria may be uncommon in children irrespective of their HIV status, using Micral test II screening method. While our data do not support the value of routine screening for microalbuminuria in HIV-infected children in Africa, future studies should determine if these results are generalizable to other HIV-infected populations. However, periodic screening can be initiated in HIV-positive children with risk factors such as older age, advanced HIV disease, HAART-naïve, and severe immunosuppression.

## Figures and Tables

**Table 1:** General characteristics of the subjects.

Parameters	Frequency (%)	*χ* ^2^	*P* value
HIV disease severity			
Advanced	6 (3.9)	116.3	<0.0001
Nonadvanced	148 (96.1)		
Use of HAART			
On HAART	119 (77.3)	45.8	<0.0001
Not on HAART	35 (22.7)		
Immunologic category			
Severe	34 (22.0)	48.0	<0.0001
Nonsevere	120 (78.0)		

**Table 2:** Mean CD4 cell count and mean CD4% of the subjects by age.

Age range	CD4 cell count ± SD	CD4 percent ± SD
	(cell/mm^3^)	(%)
18–35 months	1019.7 ± 534.2	24.0 ± 10.3
36–59 months	844.6 ± 373.4	21.2 ± 9.3
5–14 years	563.8 ± 390.4	24.4 ± 14.2

Fisher's exact (*P* value)	14.26 (<0.0001)	0.95 (0.40)

**Table 3:** Immunologic status of HIV-positive subjects for age.

Age group	Immunologic category *n* (%)		
	Severe immunosuppression	Nonsevere immunosuppression	Total
	*N* (%)	*N* (%)	*N* (%)
Below five years	23 (67.6)	43 (35.8)	66 (43.0)
Five years and above	11 (32.4)	77 (64.2)	88 (57.0)

Total	34 (100.0)	120 (100.0)	154 (100.0)

*χ* ^2^ = 10.95, *P* ≤ 0.0001.

## References

[2] UNICEF

[3] Han TM, Naicker S, Ramdial PK, Assounga AG (2006). A cross-sectional study of HIV-seropositive patients with varying degrees of proteinuria in South Africa. *Kidney International*.

[4] Ray PE, Xu L, Rakusan T, Liu X (2004). A 20-year history of childhood HIV-associated nephropathy. *Pediatric Nephrology*.

[5] Leroy B, Pressac M, Bensman A, Sinnassamy P, Courpotin C Renal status in human immunodeficiency virus infected children: a prospective study.

[6] Chaparro AI, Mitchell CD, Abitbol CL (2008). Proteinuria in children infected with the human immunodeficiency virus. *Journal of Pediatrics*.

[7] Interim WHO clinical staging of HIV/AIDS and HIV/AIDS case definitions for surveillance: African Region Reference number: WHO/HIV/2005.02. http://www.who.int/hiv/pub/guidelines/casedefinitions/en/index.html.

[8] Zambrano P, Chávez A, Chaparro X (2009). Renal compromise in HIV/AIDS in patients attended at a chilean children hospital. *Revista Chilena de Infectologia*.

[9] Esezobor C, Iroha E, Onifade E, Akinsulie A, Temiye E, Ezeaka C (2010). Prevalence of proteinuria among HIV-infected children attending a tertiary hospital in Lagos, Nigeria. *Journal of Tropical Pediatrics*.

[10] Eke FU, Anochie IC, Okpere AN, Eneh AU, Ugwu RN, Ejilemele AA (2010). Microalbuminuria in children with human immunodeficiency virus (HIV) infection in Port Harcourt, Nigeria. *Nigerian Journal of Medicine*.

[12] Vogt BA, Avner ED, Behrman RE, Kliegman RM, Jenson HB (2004). Conditions particularly associated with proteinuria. *Nelson Textbook of Paediatrics*.

[13] Marshall SM, Shearing PA, Alberti KGMM (1992). Micral-test strips evaluated for screening for albuminuria. *Clinical Chemistry*.

[14] Carter JL, Tomson CRV, Stevens PE, Lamb EJ (2006). Does urinary tract infection cause proteinuria or microalbuminuria? A systematic review. *Nephrology Dialysis Transplantation*.

[15] Brown BJ, Asinobi AO, Fatunde OJ, Osinusi K, Fasina NA (2004). Evaluation of the nitrite test in screening for urinary tract infection in febrile children with sickle cell anaemia. *Nigerian Journal of Paediatrics*.

[16] Whiting P, Westwood M, Watt I, Cooper J, Kleijnen J (2005). Rapid tests and urine sampling techniques for the diagnosis of urinary tract infection (UTI) in children under five years: a systematic review. *Pediatrics*.

[17] Parikh CR, Fischer MJ, Estacio R, Schrier RW (2004). Rapid microalbuminuria screening in type 2 diabetes mellitus: simplified approach with micral test strips and specific gravity. *Nephrology Dialysis Transplantation*.

[18] Daniel WW (2005). Estimation. *Determination of Sample Size for Estimating Proportion*.

[19] Tindyebwa D, Kayita J, Musoke P (2006). *Handbook on Paediatric AIDS in Africa*.

[20] Lepore G, Maglio ML, Nosari I, Dodesini AR, Trevisan R (2002). Cost-effectiveness of two screening programs for microalbuminuria in type 2 diabetes. *Diabetes Care*.

[21] Tindyebwa D, Kayita J, Musoke P (2006). *Handbook on Paediatric AIDS in Africa*.

[22] Baekken M, Os I, Sandvik L, Oektedalen O (2008). Microalbuminuria associated with indicators of inflammatory activity in an HIV-positive population. *Nephrology Dialysis Transplantation*.

[23] Winston JA, Bruggeman LA, Ross MD (2001). Nephropathy and establishment of a renal reservoir of HIV type I during primary infection. *The New England Journal of Medicine*.

[24] Szczech LA, Grunfeld C, Scherzer R (2007). Microalbuminuria in HIV infection. *AIDS*.

[25] Gupta SK, Parker RA, Robbins GK, Dubé MP (2005). The effects of highly active antiretroviral therapy on albuminuria in HIV-infected persons: results from a randomized trial. *Nephrology Dialysis Transplantation*.

